# Deliberate Overdose Presenting to Secondary Care: Audit of Referral, Psychiatric Follow-Up and Compliance with NICE NG225 Guidelines.

**DOI:** 10.1192/j.eurpsy.2026.11337

**Published:** 2026-07-31

**Authors:** E. R. Davies

**Affiliations:** School of Medicine, University of Liverpool, Liverpool, United Kingdom

## Abstract

**Introduction:**

Deliberate self-harm, particularly deliberate overdose, represents a frequent and high-risk mode of presentation to medical services. Prompt referral to appropriate mental health teams and thorough psychosocial assessment are crucial for mitigating future risk and improving patient outcomes.

**Objectives:**

To audit and analyse referral processes and psychiatric follow up of cases of deliberate self-harm - specifically overdose - in acute medicine at the Countess of Chester Hospital. Compliance was assessed against NICE NG225 Self harm: Assessment, Management and Preventing Recurrence (*National Institute for Health and Care Excellence 2022*) and NHS England Mental Health Access Standards (*NHS England 2021*).

**Methods:**

A retrospective review of patient records within a 6-month period from January 2024-June 2024 was undertaken. Adult patients who presented with deliberate overdose within the acute medical unit and urgent care in the Countess of Chester Hospital were selected. 29 cases were identified via electronic record screening, following exclusion, 22 patients remained. Main outcome measures included time intervals between initial presentation, referral, and assessment by liaison psychiatry; documentation of psychosocial and risk assessments/formulations; and details of management plans, including community follow-up and safe prescribing practices.

**Results:**

Mean time for referral to mental health by acute medical staff from intial presentation was 20.03 hours, 0% being referred within 1 hour. Mean time to be seen from referral was 21.53 hours, 9.09% being seen within 1 hour. Mean time taken to be seen by a suitable mental health professional from initial presentation was 41.88 hours, 0% being seen within 1 hour. Most common reason for delay included waiting for patient medical optimisation. Of 22 patients seen by a mental health professional, 63.6% had full psychosocial assessments performed and correctly documented. No patients had a risk assessment done. 63.6% had a risk formulation done. 33% had safe prescribing as part of their follow up plan where indicated.

**Conclusions:**

This study demonstrated significant delays in referral and assessment for patients presenting with deliberate overdose; with complete non-compliance to the one-hour NHS England referral standard and poor adherence to NICE NG225 guidance. Waiting for patients to be medically optimised causes lengthy delay in psychiatric care and directly opposes recommendations in the guidelines. Implementation of formal staff training to illustrate the pressing importance in expeditious referrals and assessment is recommended. Re-audit following these interventions is proposed to assess improvements in compliance and patient safety outcomes. Prompt, evidence-driven psychiatric assessment is crucial to protect patients and enhance outcomes.

**Disclosure of Interest:**

None Declared

